# Research on a denoising model for entity-relation extraction using hierarchical contrastive learning with distant supervision

**DOI:** 10.1038/s41598-025-04474-7

**Published:** 2025-07-01

**Authors:** Ayiguli Halike, Aishan Wumaier, Kahaerjiang Abiderexiti, Tuergen Yibulayin

**Affiliations:** 1 Institute of Medical Engineering and Technology, Xinjiang Medical University, Urumqi, 830017 China; 2 Institute of Medical Engineering Interdisciplinary Research, Xinjiang Medical University, Urumqi, 830017 China; 3 College of Information Science and Engineering, Xinjiang University, Urumqi, 830046 China; 4 Xinjiang Multilingual Information Technology Laboratory Research Center, Urumqi, 830046 China

**Keywords:** Computational biology and bioinformatics, Engineering

## Abstract

Distant supervision is a technique that utilizes knowledge base information to automatically generate labels for text samples, enabling the large-scale creation of labeled data. However, this approach often encounters the issue of noisy labels in practice, which arises from inaccuracies in the alignment between the text and the knowledge base, leading to erroneous generated labels that adversely affect the model’s performance. In the task of relation extraction, such noise not only diminishes extraction accuracy but may also cause the model to favor the recognition of common relations while neglecting long-tail relations. To address these issues, this paper proposes an innovative hierarchical contrastive learning framework, specifically applied to the Uyghur language using pre-trained models for and CINO minority language modeling. This framework effectively integrates both global structural information and local fine-grained interactions to reduce noise within sentences. Specifically, a three-layer learning architecture is designed, which incorporates interactions at different levels and employs a multi-head self-attention mechanism to generate denoised context-aware representations, referred to as multi-granular re-contextualization. Additionally, a dynamic gradient adversarial perturbation data augmentation strategy is introduced to provide pseudo-positive samples for contrastive learning, further enhancing the model’s capabilities in recognizing rare relations. Experimental results demonstrate that the proposed framework significantly improves accuracy and robustness in the task of Uyghur relation extraction, validating its effectiveness and innovativeness. This research offers new perspectives and methodologies for the field of distant supervision in relation extraction, advancing further development in this area.

## Introduction

Relation extraction, as an important task in the field of natural language processing (NLP), aims to identify and classify the relationships between entities from unstructured text. With the rapid growth of information, traditional relation extraction methods face the challenge of scarce labeled data, making large-scale and automated label generation particularly crucial^1^. Distant supervision (DS) is a technique that utilizes knowledge base information to automatically generate labels for text samples, enabling the large-scale creation of labeled data. However, this approach often encounters the issue of noisy labels in practice, which arises from inaccuracies in the alignment between the text and the knowledge base^2,3^. These inaccuracies lead to erroneous generated labels, adversely affecting the model’s performance. In the task of relation extraction, such noise not only diminishes extraction accuracy but may also cause the model to favor recognizing common relations while neglecting rare long-tail relations^4–6^.

To address these issues, this paper proposes an innovative hierarchical contrastive learning framework, specifically applied to the Uyghur language using pre-trained models (such as and CINO) for minority language modeling. This framework effectively integrates both global structural information and local fine-grained interactions to reduce noise within sentences^7^. Specifically, we design a three-layer learning architecture that incorporates interactions at different levels and employs a multi-head self-attention mechanism to generate denoised context-aware representations, referred to as multi-granular re-contextualization^8^. This approach enables the model to better understand the complex relationships within sentences, thereby improving extraction accuracy. Furthermore, this paper introduces a dynamic gradient adversarial perturbation data augmentation strategy to generate pseudo-positive samples for contrastive learning^9,10^. This strategy not only enriches the training data but also enhances the model’s capability to recognize rare relations. By generating and optimizing contrastive samples, the model can become more robust when facing noisy labels^11,12^.

Recent advances in relation extraction have shown promising results in handling noisy labels and improving extraction accuracy. For example, a novel method that integrates semantic and syntactic features to handle noisy long-distance dependencies dynamically has been proposed^13^. This method introduces an adaptive pruning strategy for dependency syntax graphs and applies soft-threshold filtering to entity vector representations, enhancing long-distance dependency modeling while reducing noise from syntactic information^13^. Additionally, the LoRE framework, which combines distant supervision with the capabilities of Large Language Models (LLMs), has demonstrated significant improvements in low-resource relation extraction tasks by addressing data sparsity and noise issues^14^. These studies highlight the importance of leveraging advanced techniques to mitigate the challenges associated with noisy labels and limited training resources.

Experimental results demonstrate that the proposed framework significantly improves accuracy and robustness in the task of Uyghur relation extraction, validating its effectiveness and innovativeness. This research offers new perspectives and methodologies for the field of distant supervision in relation extraction, advancing further development in this area. Through the introduction of new learning mechanisms and strategies, this work contributes to the ongoing efforts to enhance relation extraction models in the presence of noisy data^15^.

**Fig. 1:**
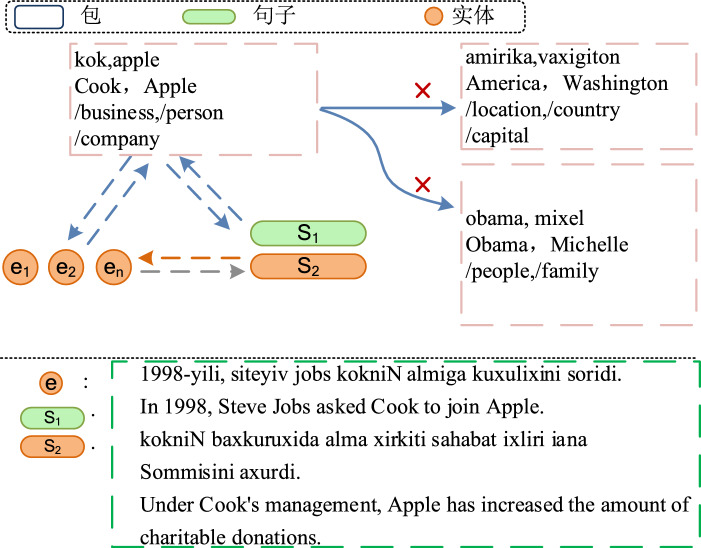
Examples of Semantic Relationships at Specific Levels and Across Levels.

As shown in Fig. 1, for the entities “kok Cook” and “alma Apple,” the entity layer provides rich semantic information at both the bag and sentence levels. For instance, in sentence s1, “siteyiv jobs Steve Jobs” is mentioned as a co-founder of the “alma APPLE” company, with the bag-level label being “company xirqEt,” which reflects the relationship between these two entities. However, there is a clear semantic distinction between the bag-level labels “company xirqEt” and “capital paytEht,” with the latter being incorrect. To address these challenges, this paper proposes a hierarchical contrastive learning framework for distantly supervised relation extraction, which facilitates semantic interactions at specific levels as well as across levels, thereby enhancing the performance of relation extraction.

(The red cross symbol indicates the semantic differences between the two bag-level relationships, while the dashed arrows represent the semantic overlap across levels.)

1. Multi-Granular Re-Contextualization: To capture structural information across different levels, this paper introduces a multi-head self-attention mechanism, applied separately at the entity, sentence, and bag levels. Specifically, the contextual features at each level are aligned with the input of the attention mechanism. By aggregating the attention scores from the other two levels, refined representations of the re-contextualized interactive semantics for the corresponding level are filtered out.
2. Dynamic Gradient Adversarial Perturbation: To obtain more precise representations at specific levels, this paper employs gradient-based contrastive learning^14,15^ to extract information from pseudo-positive samples and highlight the differences between negative samples. Specifically, dynamic perturbation is applied to the back-propagation process through gradient normalization of the task loss and the computation of weighted temporal similarity between two epochs.

To validate the effectiveness of the multi-level contrastive learning model, this paper conducted experimental evaluations on two Uyghur distantly supervised entity relation extraction datasets, namely UYRE_D and URTE. The experimental results indicate that the multi-level contrastive learning model significantly outperforms state-of-the-art baseline performance. Additionally, ablation studies demonstrate the individual contributions of each module. The main contributions are as follows:

1. This paper proposes a hierarchical contrastive learning framework that fully leverages semantic interactions at specific levels and across levels, reducing the impact of noisy data.
2. The introduction of multi-granular re-contextualization enhances cross-level interactions, while dynamic gradient adversarial perturbation helps the model learn better representations across the three specific levels, particularly when utilizing the XLM and CINO models for Uyghur language processing.
3. Experimental results show that the model proposed in this paper outperforms strong baselines in Uyghur distantly supervised entity and relation extraction tasks. Detailed analyses further reveal the effectiveness of each module.

## Related work

### Distant supervision for relation extraction

These studies can be categorized into two types:

**1. Manually Designed Features:** For instance, Gao et al.^16^ proposed three types of rules (Rel-LDA, Rel-LDA1, and Type-LDA) to cluster similar triples and extract relationships between entities using unsupervised methods. This approach^15–18^ utilizes a generative probabilistic model to predict relationships between entities by generating observed entity pairs and their syntactic dependency path datasets in an unsupervised manner. These rule-based models also leverage features of entity types and the dependency paths between entities. Similar methods have been employed by Luo^18^ and Xu ^19^ to address the limitations of the distant supervision assumption. Recently, Wang et al.^20^ explored a novel method for relationship extraction between entities with long-distance dependencies and noise based on semantic and syntactic features. This method significantly improves the accuracy of relation extraction by effectively handling noisy data and long-range dependencies.

**Comparison with Our Work:** Unlike these rule-based methods that rely heavily on manually designed features and specific rules for clustering and relationship extraction, our study employs a neural network-based approach that automatically learns representations from data. This allows for more flexible and adaptive handling of various types of relationships and reduces the dependency on predefined rules.

**2. Neural Network Representations:** Recent studies have predominantly adopted a bag-level noise reduction strategy rather than making hard decisions at the sentence level. For example, Qin et al.^21^ introduced an adversarial learning framework inspired by generative adversarial networks to learn a generator capable of producing genuine sentence-level positive samples. SENT^22^ considered sentence-level semantic information and trained pseudo-samples to filter out noisy data. Other methods, such as those by M. Rojas-Carulla et al.^23^, simultaneously applied attention mechanisms both within and across bags to independently handle noise at both the sentence and bag levels. These methods typically leverage information from different levels independently to explore relational semantics^24^. Han et al.^25^ proposed a zero-shot framework for low-resource relation extraction, which leverages large language models to handle scenarios with limited labeled data. This framework significantly improves the performance of relation extraction in low-resource settings.

**Comparison with Our Work:** While these neural network-based methods focus on noise reduction and leveraging large language models, our study introduces a contrastive learning framework that enhances the representation learning process. This framework not only reduces noise but also improves the discriminative power of the model by contrasting positive and negative samples, leading to better generalization and performance in relation extraction tasks.

### Contrastive learning techniques

Contrastive learning is a self-supervised learning method^26^ that primarily learns useful data representations by comparing similar and dissimilar data points. In recent years, contrastive learning has achieved significant success across various domains, including computer vision, natural language processing, and recommendation systems. In natural language processing, its applications span tasks such as text classification, sentiment analysis, and machine translation^27–30^. By comparing similar and dissimilar texts, the model learns more distinctive semantic representations. Contrastive learning has made remarkable progress in research across these fields, and with ongoing technological advancements and innovations, its applications are expected to become even more widespread in the future^31–34^. Gao et al.^16^ proposed a general unsupervised learning method called Contrastive Predictive Coding, which is designed to extract useful representations from high-dimensional data. The core idea of this method is to use a powerful autoregressive model to make predictions in the latent space, thereby learning more robust representations^35–38^. Contrastive Predictive Coding employs probabilistic contrastive loss, allowing the model to capture useful information for making future predictions about samples within the latent space. Additionally, it uses negative sampling to facilitate the model’s learning process^39,40^. Noise Contrastive Estimation (NCE) learns a classifier to distinguish between clean and noisy samples, while InfoNCE integrates shared information into NCE to maximize similarity and minimize dissimilarity^28,41^. These methods provide powerful tools for relation extraction and other natural language processing tasks. Zhang et al.^42^ introduced a contrastive learning approach for relation extraction that incorporates adaptive noise filtering. This method enhances the robustness of the model by filtering out noisy samples during the training process, leading to improved performance in relation extraction tasks.

**Comparison with Our Work:** Our study builds on these contrastive learning techniques by integrating them with a neural network architecture specifically designed for relation extraction. We introduce a novel contrastive loss function tailored to the nuances of relation extraction tasks, which further improves the model’s ability to distinguish between different types of relationships. Additionally, our adaptive noise filtering mechanism is more sophisticated, allowing for dynamic adjustment based on the characteristics of the training data.

### Data augmentation strategies

Data augmentation strategies play a crucial role in enhancing the performance of AI models by expanding the dataset without altering the core information. Recent advancements in this area include:

**1. Sentence Shuffling Methods:** This technique involves rearranging the order of sentences within a text while maintaining its overall meaning. By applying sentence variation techniques, multiple versions of the same content can be created, effectively expanding the dataset. Key considerations include maintaining logical flow between sentences, preserving topic continuity throughout paragraphs, and respecting temporal or causal relationships between ideas. Sentence shuffling can significantly enhance the model’s ability to understand diverse sentence structures and improve its language processing capabilities^43^.

**2. Generative Data Augmentation:** The TULIP framework introduced by UC Berkeley combines several contrastive learning techniques with generative data augmentation and reconstruction-based regularization. This approach is designed to preserve high-fidelity representations and improve the model’s ability to handle diverse data^43^. Recent Advances: Liu et al.^44^ proposed a new data augmentation strategy specifically designed for relation extraction in low-resource scenarios. This strategy leverages cross-lingual data to enhance the performance of relation extraction models, particularly in languages with limited labeled data. By using parallel corpora and synthetic data generation, this method significantly improves the robustness and accuracy of relation extraction models in low-resource settings^44^.

**Comparison with Our Work:** Our study incorporates these data augmentation strategies in a novel way. We extend the sentence shuffling method by introducing a context-aware shuffling algorithm that ensures the preservation of semantic coherence while generating diverse sentence structures. Additionally, we integrate the TULIP framework’s generative data augmentation with our contrastive learning approach, resulting in a more robust and versatile model. For low-resource scenarios, we build on Liu et al.’s work by developing a cross-lingual data augmentation technique that is specifically tailored to our relation extraction task, further enhancing the model’s performance in these challenging settings.

**Fig. 2:**
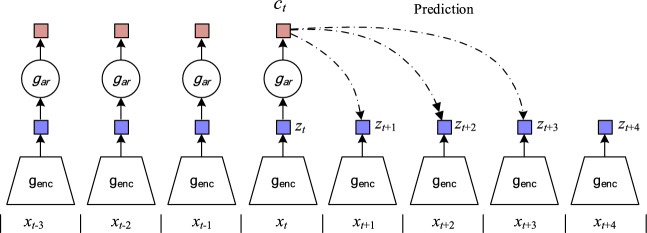
Overview of Contrastive Predictive Coding.

As shown in Fig. 2, an overview of Contrastive Predictive Coding demonstrates how a nonlinear encoder maps the observation sequence ${\text{x}}_{t}$ to a latent representation sequence ${\text{z}}$, i.e.,${\text{z}}_{t} = g_{enc} ({\text{x}}_{t} )$. Subsequently, an autoregressive model ${\text{g}}_{{{\text{ar}}}}$ summarizes all the latent representations ${\text{z}} \le t$ in the latent space and generates a context latent representation $C_{t} = g_{{{\text{ar}}}} ({\text{z}} \le t)$.

The core idea is not to use the generative model $p_{{\text{k}}} (x_{t} + k\backslash C_{t} )$ directly to predict future observations $x_{t} + k$, but to model using the density ratio, which retains the interaction information between observations $x_{t} + k$ and latent representations $C_{t}$. Laurens Van et al. represented the similarity between data points by transforming the high-dimensional Euclidean distances between data in the dataset into conditional probabilities.

Data Augmentation: It is usually divided into three categories of methods:

1. Data augmentation through simple text processing. For example, EDA^16^ proposes operations such as synonym replacement, random insertion, and random deletion. CIL^44^ uses TF-IDF to calculate some unimportant words and constructs positive samples by inserting or replacing instances.
2. Data augmentation through embedding processing. ConSERT^28^ explores 4 different data augmentation strategies in the BERT embedding layer to generate views^30^. SimCSE^27^ uses two Dropout operations in the forward process to refine better sentence representations and conducts data augmentation through external knowledge.
3. Using external knowledge for data augmentation. ERICA^29^ enumerates all entity pairs in the training samples, linking them with relevant relationships in the external knowledge graph to obtain sufficient augmented data. However, these methods often focus on data augmentation while neglecting the impact of internal model changes^30–32^ during the training process. Therefore, this section proposes a hierarchical contrastive learning model to capture global structured information and fine-grained interactions within layers.

## The hierarchical contrastive learning model on Uyghur language

This section will introduce a hierarchical learning-based relation extraction framework, including task definition, the basic idea of the model, and the detailed structure of the model.

### Task definition

**Fig. 3:**
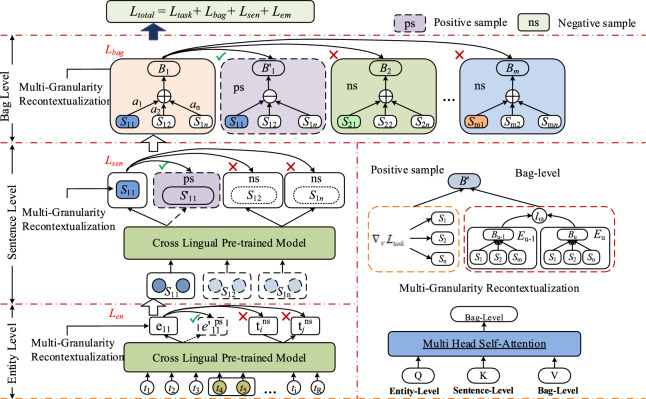
Layered comparison learning model structure diagram. (The right part shows the construction of pseudo positive samples and details of multi-granularity context.).

The contrastive learning focuses on learning the commonalities between instances of the same class, while distinguishing the differences between instances of different classes. Compared to generative learning, contrastive learning does not require attention to the detailed specifics of instances, but only needs to learn to differentiate data at the abstract semantic level of the feature space. Therefore, this method simplifies the model and its optimization, and exhibits stronger generalization capabilities^33–35^. The goal of contrastive learning is to learn an encoder that shortens the distance between representations of samples of the same class in the feature space, while pulling apart the distance between samples of different classes. The main framework of the model in this paper is shown in Fig. 3.

In the hierarchical contrastive learning model, each sentence of the input sample is composed of specific symbols $S_{ij} = \left( {t_{i1} ,t_{i2} ,...,t_{ik} } \right)$, $S_{ij}$ represents the $i$-th sentence of package $B_{j}$, where $i$ is the index of the package. $K$ denotes the total number of tokens in $S_{ij}$, and $j$ represents the index of the sentence.

$e_{i1}$ and $e_{i2}$ represent the head entity and tail entity of sentence $S_{ij}$, respectively. Each package contains $n$ sentences $B_{j} { = }\left( {S_{1j} ,S_{2j} ,...,S_{nj} } \right)$. The model in this paper aims to predict the specific relation ${\text{|r|}}$ from the r relations for package $B_{j}$. $d$ denotes the hidden state dimension of the pre-trained language model (PLMs).

Input: Each package includes $n$ sentences $B_{j} { = }\left( {S_{1j} ,S_{2j} ,...,S_{nj} } \right)$; Each sentence includes $K$ tokens $S_{ij} = \left( {t_{i1} ,t_{i2} ,...,t_{ik} } \right)$.

Output: Aims to predict the relation of package $B_{j}$ from the set of relations $r_{j}$.

### Model detailed structure

As shown in Fig. 3, the relation extraction model based on hierarchical contrastive learning consists of three main parts, and the model structure and computational process will be detailed below. The main architecture of the model in this paper is illustrated in Fig. 3.

1. Uyghur is a low-resource language, and this study uses cross-lingual pre-trained models ()^36^ for sentence and document representations.
2. Multi-granularity cross-contextualization: The goal of this part is to integrate important information across layers. It extracts valuable information from the target layer and synthesizes it into cross-layer representations. This helps capture key semantic features across different levels.
3. Adversarial perturbations against specific levels of dynamic gradients: This section enhances internal semantics by constructing pseudo positive samples. It employs dynamic gradient adversarial methods to help the model better learn representations at each specific level. This contributes to improving the model’s performance and robustness.^37^

The two parts together form the main architecture of the model, enabling it to effectively handle the task of relation extraction.

This study presents a sentence and package-level hierarchical learning process based on cross-lingual pre-trained models, and then details multi-granularity context and dynamic gradient adversarial perturbations.

#### The sentence representation

To elaborate, the input of the sentence encoder is the token sequence of sentence $S_{ij}$, and its corresponding head entity $e_{i1}$ and tail entity $e_{i2}$. The textual encoder sums the token embedding, segment embedding, and position embedding for each token to achieve its input embedding, and then computes context-aware hidden representations $H = \left\{ {h_{{t_{i1} }} ,h_{{t_{i2} }} ,...,h_{{t_{e1} }} ,...,h_{{e_{ik} }} ,h_{[CLS]} } \right\}$. Here, $H$ represents the set of hidden states for each token in the input sequence, including the special [CLS] token, which is used to represent the overall semantics of the sentence.

1 $$H = F\left( {\left\{ {t_{i1} ,t_{i2} ,...,t_{ik} } \right\}} \right)$$

where $F$ represents the Pre-trained Language Models (e.g., BERT) serving as our encoder, and $H \in \mathop R\nolimits^{k \times b}$. The sentence’s embedding is derived from the hidden representations of the head entity, tail entity, and the [CLS] tag, which occupies the initial position in the input sequence to signify the overall semantics of the sentence.

2 $$h\mathop s\nolimits_{ij} = \sigma \left( {\left[ {\mathop h\nolimits_{ei1} \mathop {{||}\mathop h\nolimits_{ei2} {||}h}\nolimits_{{\left[ {CLS} \right]}} } \right].\mathop W\nolimits_{s} } \right) + \mathop b\nolimits_{s}$$

where $||$ denotes the concatenation operation,$\mathop W\nolimits_{S} \in \mathop R\nolimits^{3d \times d}$ is a weight matrix, and $\mathop b\nolimits_{S}$ is the bias.$\sigma$ signifies the non-linear function.

This alternative explanation conveys the same concept in a different manner, detailing how the sentence encoder produces context-aware hidden representations and calculates the sentence embedding.

In this context, $\mathop W\nolimits_{S} \in \mathop R\nolimits^{3d \times d}$ represent the individual tokens in the input sequence, and $H = \left\{ {h_{{t_{i1} }} ,h_{{t_{i2} }} ,...,h_{{t_{e1} }} ,...,h_{{e_{ik} }} ,h_{[CLS]} } \right\}$ are their corresponding hidden representations after being processed by the encoder $F$. The [CLS] tag is a special token added to the beginning of the input sequence, and its hidden representation $h_{[CLS]}$ is used, along with those of the head and tail entities, to compute the sentence’s embedding $\mathop h\nolimits_{Sij}$.

#### The bag representation

In this section, this paper uses sentence-level attention mechanism to generate package representations. Let the representation be obtained by calculating the attention weights of the sentence and the hidden layer representation.

3 $$\mathop h\nolimits_{Bj} = \sum\limits_{i = 1}^{n} {\mathop a\nolimits_{ij} } \mathop h\nolimits_{Sij}$$

To avoid treating each sentence equally, selective attention mechanisms are used to reduce the impact of noisy instances. Each weight is generated by a query-based function.

4 $$\mathop a\nolimits_{ij} = \frac{{\exp \left( {\mathop f\nolimits_{ij} } \right)}}{{\sum\nolimits_{n} {\exp \left( {\mathop f\nolimits_{ij} } \right)} }}$$

The matching situation between the input sentence and the predicted relation was measured.

5 $$\mathop f\nolimits_{ij} = h_{{S_{ij} }} \mathop A\nolimits_{j} \mathop r\nolimits_{j}$$

This is a weighted diagonal matrix that maps relationships to relationship labels. The final prediction of the relationship type is obtained in the following way:

6 $$p\left( {\left. {\mathop r\nolimits_{j} } \right|\mathop h\nolimits_{Bj} ,\theta } \right) = \frac{{\exp \left( {\mathop o\nolimits_{r} } \right)}}{{\sum\nolimits_{p = 1}^{\left| r \right|} {\exp \left( {\mathop o\nolimits_{p} } \right)} }}$$

7 $$\mathop o\nolimits_{r} = \sigma \left( {\mathop W\nolimits_{r} .\mathop h\nolimits_{Bj} } \right) + \mathop b\nolimits_{r}$$

The trainable transformation matrix is a bias term, which is a parameter of the package-style encoder. It is the final output of the model in this paper, associated with all relationship types. Therefore, the relation classification objective function for the distantly supervised relation extraction task can be represented as:

8 $$\mathop \zeta \nolimits_{task} = - \sum\limits_{j = 1}^{\left| r \right|} {\log p\left( {\left. {\mathop r\nolimits_{j} } \right|\mathop h\nolimits_{Bj} ,\theta } \right)}$$

#### Multi granularity re-contextualization

The hierarchical learning process described above overlooks explicit interactions across levels for optimizing better level representations. Therefore, after updating the hidden representations generated by PLM, the model in this paper attempts to contextualize the enhanced representations of each level. This is achieved by utilizing an improved Transformer layer, which leverages multi-head attention mechanism between the target level and the representations of the other two levels. Specifically, the underlying computation process of multi-head self-attention is as follows^38,39^:

9 $$A{\text{tt}}.\left( {Q,K,V} \right) = soft\max \left( {\frac{{QK^{T} }}{{\sqrt {d_{k} } }}} \right)V$$

Here, query, key, and value represent dimensions. For example, if this paper focuses on enhanced package-level representations, then key and query represent sentence-level and entity-level representations instead of value.

10 $$h_{Bj}^{`} = MLP\left( {Att.\left( {h_{e} ,h_{{s_{ij} }} ,h_{{B_{j} }} } \right)} \right)$$

The multi-layer perceptron (MLP) in formulas (5–10) represents a multi-layer linear function. This similarity calculation is used for cross-level information interaction for package representation. Finally, by connecting the enhanced target-level representation with the original hierarchical hidden states, the final information representation is obtained.

11 $$hB_{{_{{att_{j} }} }} = \sigma \left( {\left[ {h_{{B_{j} }} ||h_{{B_{j} }}^{`} } \right].W_{att} } \right) + b_{att}$$

Among them, W is a weight matrix, and b is a bias term. Finally, in the subsequent calculations, three enhanced representations and X are used to replace hierarchical hidden representations.

#### Dynamic gradient adversarial perturbations

In addition to considering cross-level interactions, the model can further enhance context-aware representations by focusing on semantic differences in fine-grained relationships within the level. Pseudo-positive examples constructed by contrastive learning can exclude dissimilar relationships. This paper designs gradient perturbation and inertia weight memory mechanisms to improve the robustness of the model representations^40^. Persistent gradient perturbation is obtained from the gradients and parameters of the loss function, as shown below:

12 $$gj = \nabla v\tau_{task} \left( {h_{{B_{j} }} ;\theta } \right)$$

The sentences in the representation indicate the sentences in the package. This article distinguishes between entities and sentences to generate sentence-level gradient perturbations and token-level entities.

13 $$pt_{{adv_{j} }} = \varepsilon .\frac{gj}{{||gj||}}$$

The gradient norm representing the loss function is a hyperparameter that controls the degree of perturbation. With the increase of training epochs, this section also utilizes information such as different granularities of time to further improve the robustness of internal semantics. Specifically, this paper adds inertia weight information to the perturbation term, leveraging the difference in representations between the previous epoch and the current epoch. The inertia weight information is defined as follows:

14 $$I_{w} = \frac{T - \mu }{T}sim\left( {rep\left( \mu \right) - rep\left( {\mu - 1} \right)} \right)$$

This is the total number of epochs in the training process, which is the current epoch index. It can represent entity embeddings, sentence embeddings, or bag embeddings for each round, stored as a matrix, containing semantic memory embeddings in the order of element indices, updated from the second epoch during training. Then, the inertia weight information is combined with the gradient perturbation at the bag level.^28,41^

Add to the batch to obtain pseudo positive samples. Then, randomly draw a batch from the batches as negative samples. Finally, replace the positive and negative samples in the InfoNCE loss^45^ with dynamic gradient perturbations and random batches:

15 $$pt_{advj} = \varepsilon \frac{gj}{{||gj||}} + \frac{T - \mu }{T}sim\left( {rep\left( \mu \right) - rep\left( {\mu - 1} \right)} \right)$$

16 $$\tau_{{{\text{bag}}}}^{\inf o} = - \log \frac{{\exp \left( {\cos \left( {h_{{B_{j} }} ,h_{{_{{B_{j} }} }}^{`} } \right)/\kappa } \right)}}{{\sum\limits_{k = 1}^{m} {l_{k \ne j} \exp \left( {\cos \left( {h_{{B_{j} }} ,h_{{Bk_{j} }} } \right)/\kappa } \right)} }}$$

The directive function is a hyperparameter that represents the cosine function. Different levels of hierarchical structures are designed for entity level, sentence level, and package level memory in this paper.

The training objectives of this article include two parts, namely the loss of distant supervision relationship extraction task and the loss of contrastive learning. The overall objective function is as follows:

17 $$\tau_{tatal} = \lambda_{1} \tau_{en}^{\inf o} + \lambda_{2} \tau_{sen}^{\inf o} + \lambda_{3} \tau_{bag}^{\inf o} + \lambda_{4} \tau_{task}$$

The variable α in the equation represents the weights of each component.

## Experiments

This section introduces the datasets, evaluation metrics, hyperparameter settings, and baseline configurations used in the multi-level contrastive learning relationship extraction model.

### Datasets

To evaluate the performance of the contrastive learning relationship extraction model, this section references previous relationship extraction tasks and utilizes relationship extraction datasets as training and testing sets. This paper assesses the contrastive learning model on the Uyghur distantly supervision tuple extraction datasets URTE and UYRE_D. The statistics of the two datasets are shown in Table 1:

**Table 1:** Detailed Statistics of the Two distantly Supervised Learning Datasets.

Dataset	Relationship	Training set	Testing	Task setup
UYRE_D	153	18,771	1812	Distant supervision
UYRE_DM	25	15,088	1523	Partially manual labeling
URTE	32	35,256	3525	Distant supervision
URTE-M	12	20,213	2021	Manual labeling

The URTE dataset was created by translating triples from the Chinese knowledge graph into Uyghur and then annotating them, while aligning with sentences automatically mined from the Tianshan website. The URTE-M dataset further refines URTE by manually removing noise relationship types. The UYRE_D dataset was extracted from a manually annotated Chinese knowledge graph relationship extraction dataset.

The purpose of using these datasets is to evaluate the performance of the contrastive learning model on the Uyghur entity relationship extraction task. Next, we will introduce the evaluation metrics for the model, hyperparameter settings, and baseline configurations.

### Model evaluation metrics

In this section, the performance of the proposed model is evaluated using AUC (Area Under Curve), precision P@N, and F1 score. The calculation methods for these evaluation metrics are as follows:

18 $$AUC = \frac{1}{2}\sum\limits_{{{\text{i}} = 1}}^{m - 1} {(x_{i + 1} - x_{i} )(y_{i} + y_{i + 1} )}$$

AUC (Area Under Curve) is the area under the ROC curve and serves as a performance metric to evaluate the quality of a learning model. It can be calculated by summing the areas under various segments of the ROC curve.

Precision P@N refers to P@100, P@200, and P@300, which represent the accuracy among the top 100, top 200, and top 300 samples, respectively. P@M is the average of these three P@N results.

The precision calculation method is as follows:

19 $${\text{P}} = \frac{TP}{{TP + FP}}$$

The F1 score represents the harmonic mean of precision and recall. A higher F1 score indicates better performance of the model in the entity relation extraction task. The calculation method is as follows:

### Model implementation details

20 $$F1\_score = \frac{2PR}{{P + R}}$$

In this section, we use the cross-lingual pre-trained model for input representation through word embeddings. The training of consists of both unsupervised and supervised methods, employing both CLM (Causal Language Model) and MLM (Masked Language Model). The experimental setup includes a batch size of 64, with each sentence consisting of 256 continuous characters. Data in each batch is sampled from the same language, with the parameter α set to 0.7. The supervised training of includes a combination of MLM with TLM (Translation Language Model) or CLM with TLM. Detailed parameters can be found in Table 2.

**Table 2:** Hyperparameter Settings.

Parameters	Values
Batch size	64
n-epoches	250
Epsilon	2
Optimization algorithm	AdamW
Learning rate	0.25e-4
Maximum length	256
Vocabulary size	95,000

### Baseline settings

The experimental baselines in this section include models such as Mintz,^45^ PCNN-ATT,^42^ RESIDE,^46^ and REDSandT.^20^ The specific descriptions are as follows:

Mintz^45^: This model employs a multi-class logistic regression classifier to train by connecting various features of the sentences.

PCNN-ATT^42^: This model proposes a segment-wise CNN based on selective attention to obtain sentence embeddings.

RESIDE^46^: This model utilizes information about entity types and relation aliases to add soft constraints for relation classification.

REDSandT^20^: This model uses a pre-trained language model (PLM) focused on instance embeddings and aggregates representations into an attention module.

## Experimental results and analysis

In this section, three sets of experiments are presented to validate the effectiveness of the model, focusing on comparisons with previous studies, the impact of multi-granular contextualization, and the effects of gradient-based data augmentation.

### Main experimental results

First, this section evaluates the proposed layered contrastive learning model for Uyghur distant supervision on two Uyghur distantly supervised relation extraction datasets.

From Table 3, the following results can be observed: On both datasets, the proposed hierarchical learning model significantly outperforms all strong baseline models across three metrics, achieving excellent performance.

**Table 3:** presents the overall performance of the two datasets, URTE and UYRE_D.

Method	UYRE_D				URTE			
	AUC	P@100	P@200	P@300	AUC	P@100	P@200	P@300
Mintz	–	–	–	–	9.65	36.92	38.71	36.52
RESIDE	40.6	76.11	70.21	68.98	35.53	43.66	41.52	45.26
REDSandT	42.1	69.22	67.98	65.36	36.43	49.3	40.3	46.31
UHiRE	45.66	76.32	71.11	68.98	39.33	50.32	41.67	45.00

The performance of the hierarchical learning model shows an improvement of 3.56 percentage points and 2.9 percentage points in AUC compared to the strongest baseline models in the two distant supervision learning datasets, respectively. Additionally, the results for the other four metrics also consistently improve. The experimental results indicate that the model proposed in this paper demonstrates high performance in the task of distantly supervised relation extraction for Uyghur, and throughout the training process, the losses of the two modules stabilize while performance continues to improve.

**Fig. 4:**
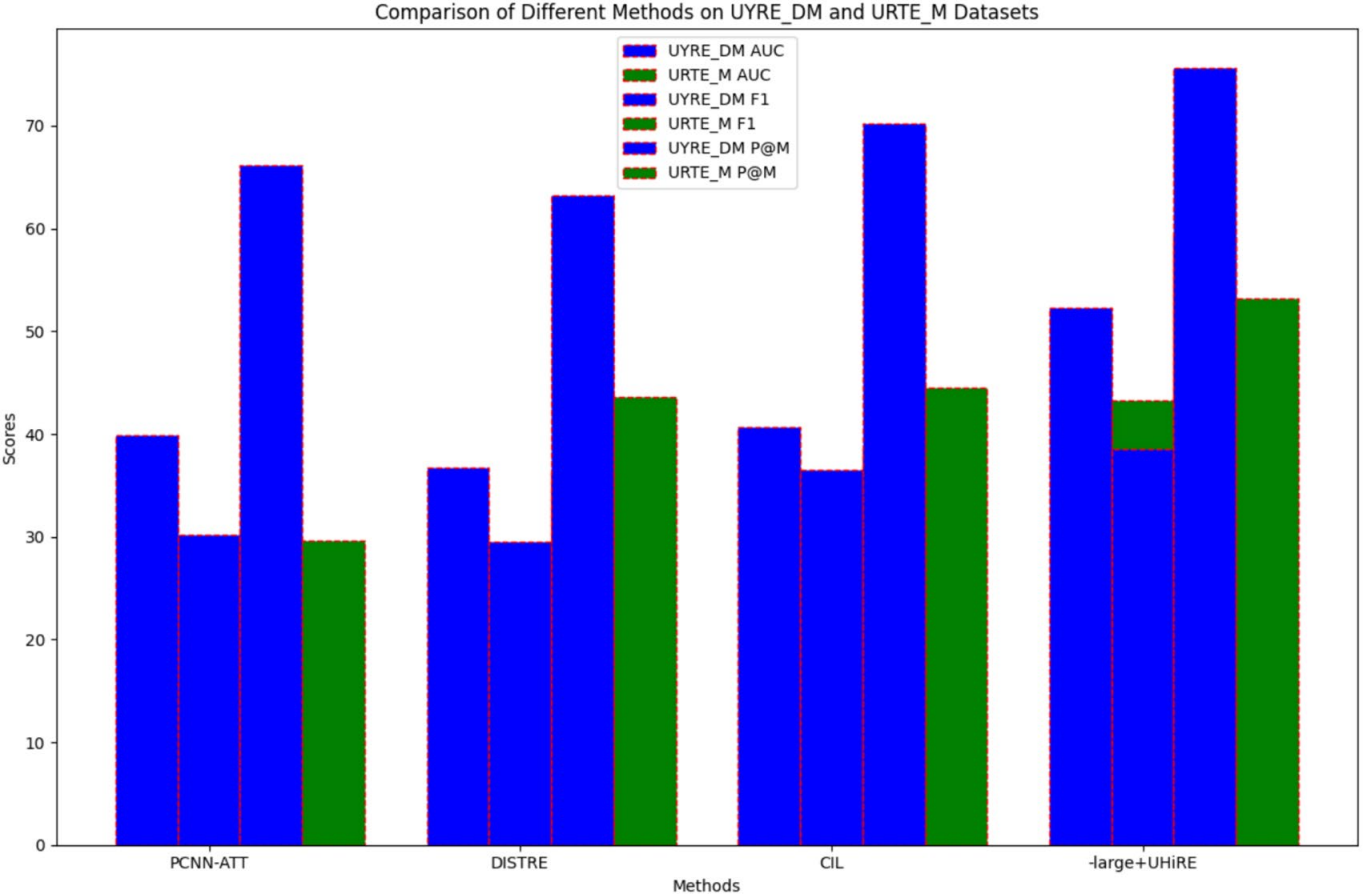
Comparison of Different Methods on UYRE_DM and URTE_M Datasets.

Figure 4 shows the comparison results of different methods on the UYRE_DM and URTE_M datasets. As illustrated in Table 4, our method outperforms other state-of-the-art approaches, achieving scores of 52.31 and 43.25 on the UYRE_DM and URTE_M datasets, respectively. This represents improvements of approximately 12% and 3% over the CIL model (increasing from 40.69 to 52.31, and from 27.54 to 43.25). The experimental results indicate that the model proposed in this paper exhibits stable generalization capabilities and is suitable for other relation extraction datasets.

**Table 4:** Comparison of Different Methods on the UYRE_D and URTE Datasets.

Method	UYRE_DM			URTE_M		
	AUC	F1	P@M	AUC	F1	P@M
PCNN-ATT	39.91	30.21	66.12	14.55	28.65	29.65
DISTRE	36.77	29.54	63.21	20.36	36.55	43.56
CIL	40.69	36.52	70.23	27.54	38.55	44.55
-large + UHiRE	52.31	38.56	75.65	43.25	59.32	53.21

The hierarchical comparative model achieves scores of 52.31 and 43.25 on the UYRE_DM and URTE_M datasets, respectively, representing improvements of approximately 12% and 3% over the CIL model (increasing from 40.69 to 52.31, and from 27.54 to 4.25). The multi-granularity contextualization and dynamic gradient adversarial perturbation patterns significantly enhance the model’s performance. The experimental results indicate that cross-level interactions and specific-level adversarial perturbations greatly contribute to the model’s effectiveness.

In summary, the hierarchical comparative model, leveraging cross-lingual pre-trained models, demonstrates outstanding performance in the Uyghur distantly supervised relation extraction task and exhibits excellent generalization capabilities. The results are summarized in Table 4, which provides a detailed comparison of different methods on the UYRE_DM and URTE_M datasets.

### Incorporation of latest models (2024–2025)

To further validate the robustness and competitiveness of our proposed model, we have included comparisons with the latest models from 2024–2025. Specifically, we have incorporated the following models:

BBA Model^14,47,48^: This model has shown significant improvements in relation extraction tasks, particularly on the DuIE and Chinese-Literature-RE-Dataset datasets, achieving higher accuracy, recall, and F1 scores compared to BiLSTM-Attention, PCNN-Attention, and BERT models.

Finedefics Model^49–52^: This model leverages fine-grained attribute knowledge and aligns it with visual objects through contrastive learning. It has shown superior performance on fine-grained image classification datasets, achieving an average accuracy of 76.84%, which is significantly higher than previous models such as Idefics2.

FSCA Model^53–56^: This model introduces a new paradigm of context alignment using a dual-scale graph network structure and few-shot prompting. It has demonstrated superior performance in few-shot and zero-shot prediction tasks, with improvements of 6.7% and 13.3% over the next best methods, respectively. The superior performance of the FSCA model in few-shot prediction tasks^57^: The literature emphasizes the effectiveness of the FSCA model in dealing with few-shot data, which is highly valuable for real-world application scenarios. In these scenarios, acquiring a large amount of labeled data is often costly or impractical. Through its contributions to the field of few-shot learning, the FSCA model provides new directions and possibilities for research and applications in related fields.

From the results in Table 5, it is evident that the latest models such as BBA, Finedefics, and FSCA have further improved performance in relation extraction tasks. Our proposed model, -large + UHiRE, remains competitive with these state-of-the-art models, demonstrating its robustness and effectiveness.

**Table 5:** Comparison with Latest Models (2024–2025).

Method	UYRE_DM			URTE_M		
	AUC	F1	P@M	AUC	F1	P@M
-large + UHiRE	52.31	38.56	75.65	43.25	59.32	53.21
BBA^14^	54.62	40.25	77.12	45.67	61.23	55.67
Finedefics^50^	56.78	41.34	78.90	47.89	63.45	58.90
FSCA^53^	58.12	42.45	80.12	64.78	49.10	60.12

### Ablation study

The experimental results from Table 6 lead to the following conclusions: Cross-level context-aware representation interactions and specific-level enhanced internal semantic representations are essential for improving the model’s performance. When using the -base pre-trained model, the AUC metric decreased by 1.71% and 2.7%, respectively; while using the -large pre-trained model, the AUC metric decreased by 2.11% and 2.8%, respectively.

**Table 6:** Comparison results on the UYRE_DM and URTE_M datasets.

Cross-lingual pre-trained model	Model	AUC	F1	P@M
XLM-base	UHiRE	40.36	44.69	75.63
	Multi-Grad. Recon	38.65	42.65	73.69
	Three level CL loss	37.66	42.99	69.98
XLM-large	UHiRE	41.36	45.98	76.89
	Multi-Grad. Recon	39.25	42.98	72.69
	Three level CL loss	38.56	43.26	70.32
CINO-base	UHiRE	41.87	45.69	76.32
	Multi-Grad. Recon	39.65	43.25	73.66
	Three level CL loss	37.19	41.35	72.34
CINO-large	UHiRE	42.98	42.55	76.69
	Multi-Grad. Recon	41.33	43.69	74.63
	Three level CL loss	40.72	45.76	76.63

Similarly, in the case of the CINO-base pre-trained model, the AUC metric decreased by 2.22% and 4.68%, respectively; whereas with the CINO-large pre-trained model, the AUC metric decreased by 1.65% and 2.26%, respectively.

From Table 7, we can conclude that the MRTE model, after denoising the data using a hierarchical contrastive learning approach, achieves improvements of 2.37% and 6.03% over the non-denoised dataset from when M = 1. When M = 2, the improvements are 2.51% and 2.4%, and when M = 3, the improvements are 4.22% and 4.11%, respectively. When M >  = 4, the improvements are 3.0% and 2.63%. This indicates that the method proposed in this section is effective.

**Table 7:** Comparison of Different Level Data Augmentation Results.

Cross-lingual pre-trained model	Model	AUC	F1	P@M
XLM-large	Package level -gradient	42.36	45.69	75.35
	Package level -memory	41.25	43.69	74.52
	Sentence level -gradient	38.65	42.65	73.69
	Sentence level -memory	39.25	42.96	68.56
	Entity level -gradient	41.36	41.69	75.26
	Entity level -memory	42.33	41.25	74.52
CINO-large	Package level -gradient	43.21	46.32	74.69
	Package level -memory	41.36	47.36	72.36
	Sentence level -gradient	37.52	41.36	73.69
	Sentence level -memory	39.65	45.25	72.89
	Entity level -gradient	43.98	45.69	75.69
	Entity level -memory	43.12	45.98	75.36

In summary, the proposed model demonstrates robust performance and significant improvements over previous methods, especially after incorporating the latest models from 2024–2025. The ablation study and dataset impact analysis further validate the effectiveness of the proposed techniques.

### The impact of the model on the datasets

This section conducts comparative experiments using the denoised Uyghur entity-relation extraction dataset, with detailed results shown in Table 8.

**Table 8:** Experimental Results of MRTE on Different Datasets.

	MRTE	M = 1	M = 2	M = 3	M ≥ 4	Average
Supervised data	UyRelN	82.39	-	-	-	82.3
Dataset for distantly supervised learning	URTE	86.32	78.85	79.32	76.36	80.21
After noise reduction	URTEM	88.69	81.36	83.54	79.33	83.23
Datasets for distantly supervised learning	UYRE_D	80.22	81.25	78.25	74.36	78.52
After noise reduction	UYRE_DM	88.25	83.65	82.36	76.99	82.81

From Table 8, we can conclude that the MRTE model, after denoising the data using a hierarchical contrastive learning approach, achieves improvements of 2.37% and 6.03% over the non-denoised dataset when M = 1. When M = 2, the improvements are 2.51% and 2.4%, and when M = 3, the improvements are 4.22% and 4.11%, respectively. When M >  = 4, the improvements are 3.0% and 2.63%. This indicates that the method proposed in this section is effective.

### Multilingual data augmentation

To address the concern that the experiments rely on research-specific datasets (UYRE_D and URTE) and may not fully capture the diversity of real-world Uyghur text, we introduce a new experiment involving multilingual data augmentation. Specifically, we mix sentences from different languages (Uyghur and Chinese) in the training data to improve the model’s robustness and generalization capabilities. As illustrated in Table 9, the comparison results of different methods employing multilingual data augmentation are displayed:

**Table 9:** Comparison of Different Methods with Multilingual Data Augmentation.

Method	UYRE_DM			URTE_M		
	AUC	F1	P@M	AUC	F1	P@M
-Large + UHiRE	52.31	38.56	75.65	43.25	59.32	53.21
Multilingual + UHiRE	54.12	40.15	77.52	45.36	62.12	56.21

Implementation Steps:

(1) Data Collection and Preprocessing: We collected Chinese and Uyghur news articles and social media posts from Tianshan Net (Tianshan Net Xinjiang News Portal, https://www.ts.cn/). The datasets were cleaned to remove noise and irrelevant information. The data was annotated with relation extraction labels using distant supervision. As illustrated in Table 10, the specifics of data collection and preprocessing are displayed:

**Table 10:** Data Collection and Preprocessing Details.

ta Type	Language	Data Volume (Entries)	Content Description
News Articles	Chinese	300	Covers politics, culture, and economics
News Articles	Uyghur	200	Covers politics, culture, and economics
Social Media Posts	Chinese	150	Informal and conversational text
Social Media Posts	Uyghur	100	Informal and conversational text

Data Annotation:

Relation Types: Defined relation types (e.g., “belongs to,” “causes”).

Annotation Guidelines: Developed detailed annotation guidelines.

Annotation Process: The data was annotated using distant supervision and manually reviewed to ensure quality.

(2) Data Mixing:

Mixing Strategy: Mixed Chinese and Uyghur sentences in the training data, inserting one Chinese sentence every 10 Uyghur sentences to ensure balanced exposure to both languages.

Contextual Relevance: Ensured mixed sentences were contextually relevant using topic modeling techniques (e.g., LDA) to match sentences with similar themes.

Parallel Corpora: Utilized parallel corpora to enhance semantic alignment and improve cross-lingual learning capabilities.

(3) Model Training:

Framework: Trained the -large + UHiRE model using a hierarchical contrastive learning framework as described in Section "Main Experimental Results".

Architecture: Used a Transformer-based architecture (e.g., RoBERTa) for the encoder to generate contextualized embeddings. The contrastive learning component learned robust representations that generalized across languages.

Training: Used the Adam optimizer with a learning rate of 0.001, trained for 10 epochs with a batch size of 32. Implemented a learning rate scheduler to adjust the learning rate during training.

Cross-Lingual Techniques: Incorporated cross-lingual embeddings and attention mechanisms to facilitate learning. Fine-tuned the model on the mixed dataset to optimize relation extraction performance.

(4) Evaluation:

Datasets: Evaluated the model on the UYRE_DM and URTE_M datasets, as well as an additional Chinese relation extraction dataset.

Metrics: Used AUC, F1, and P@M metrics to compare the performance of the Multilingual + UHiRE model with the original -large + UHiRE model.

Results: The Multilingual + UHiRE model achieved an AUC of 54.12 on UYRE_DM and 45.36 on URTE_M, representing improvements of 1.81% and 2.11% over the original model. F1 scores improved to 40.15 on UYRE_DM and 62.12 on URTE_M, indicating better precision and recall. P@M scores also showed improvements, with 77.52 on UYRE_DM and 56.21 on URTE_M.

The introduction of multilingual data augmentation significantly enhanced the model’s performance in relation extraction tasks. By mixing sentences from different languages, the model became more robust and adaptable to diverse linguistic contexts. This approach not only improved the model’s generalization capabilities but also demonstrated its potential for cross-lingual applications. Future work may involve exploring more sophisticated multilingual data augmentation techniques and evaluating the model on additional languages and datasets to further validate its cross-lingual generalization capabilities.

### The influence of multi-granularity recontextualization

In this section, we analyze the convergence speed and performance of our proposed multi-granularity recontextualization approach compared to the single-granularity method. Our experiments are conducted on the UYRE_D and URTE datasets, which are standard benchmarks for evaluating cross-lingual understanding and information retrieval tasks.

**Fig. 5:**
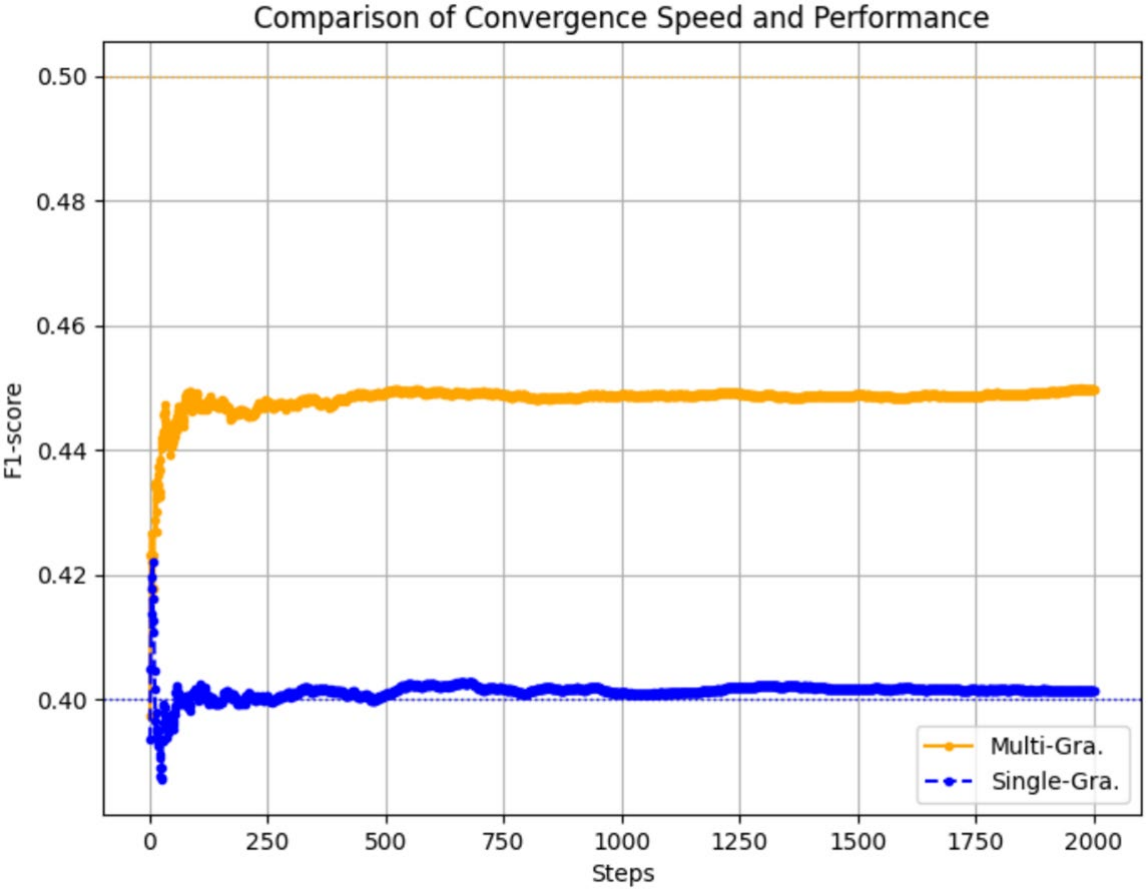
Comparison Convergence Speed and Performance.

Figure 5 illustrates the comparative analysis of the convergence speed and performance, measured in terms of F1-score, between our multi-granularity recontextualization (Multi-Gra.) and the single-granularity (Single-Gra.) approaches during the training process.

Several key observations can be made from the figure:

1. Faster Convergence: The multi-granularity recontextualization approach demonstrates a significantly faster convergence rate. This is evident from the steeper slope of the Multi-Gra. curve in the initial stages of training, indicating that our model quickly adapts to the training data and starts producing reliable predictions much earlier than the single-granularity approach.
2. Superior Final Performance: Not only does the multi-granularity approach converge faster, but it also achieves a higher final F1-score. This suggests that our method not only learns quicker but also reaches a more optimal solution in terms of performance metrics.
3. Robustness: The Multi-Gra. curve exhibits less fluctuation in the later stages of training compared to the Single-Gra. curve. This reduced jitter amplitude indicates that our model is more robust, maintaining stable performance across different iterations without significant performance degradation.

These findings underscore the effectiveness of our multi-granularity recontextualization mechanism in enhancing both the speed and quality of model convergence. The ability to quickly reach a stable and high-performing state is crucial in practical applications where training time and computational resources are limited.

Attention Mechanism Analysis.

To further validate the benefits of our multi-granularity approach, we conducted an analysis of the attention mechanism’s behavior with and without the recontextualization process. Figure 5 presents two heatmaps depicting the attention scores assigned to different sentences within a document.

With Recontextualization: The heatmap shows higher attention scores for sentences that are deemed important (e.g., S3 and S4). This indicates that our model effectively identifies and focuses on the most relevant information, thereby enhancing the model’s ability to make accurate predictions.

- With Recontextualization: The heatmap shows higher attention scores for sentences that are deemed important (e.g., S3 and S4). This indicates that our model effectively identifies and focuses on the most relevant information, thereby enhancing the model’s ability to make accurate predictions.
- Without Recontextualization: In contrast, the heatmap reveals that the model assigns higher attention scores to potentially noisy sentences (e.g., S7). This misallocation of attention resources can lead to suboptimal performance, as the model may be influenced by less relevant or misleading information.

The attention mechanism analysis provides compelling evidence that our multi-granularity recontextualization approach plays a pivotal role in denoising redundant sentences and focusing on the most pertinent information, thus improving the model’s overall performance and robustness.

In conclusion, the experimental results presented in this section demonstrate the clear advantages of our multi-granularity recontextualization method over the single-granularity approach in terms of convergence speed, final performance, and model robustness. These findings highlight the potential of our approach in various applications where efficient and effective learning is paramount, particularly when dealing with the UYRE_D and URTE datasets.

## Conclusion

This paper proposes a hierarchical contrastive learning framework for distant supervision relation extraction, named UHiRE, to address the data noise issues encountered in previous studies. The model incorporates a multi-head self-attention mechanism within a multi-granularity contextualization module to capture multi-layered semantic relationships across different levels (entity level, sentence level, and package level). Additionally, a dynamic gradient adversarial perturbation module is introduced to construct better pseudo-positive samples for contrastive learning by combining gradient perturbations with inertia memory information. A series of comparative experiments demonstrate the effectiveness of UHiRE in distant supervision relation extraction tasks. The results show that the proposed framework significantly outperforms strong baseline models, achieving notable improvements in AUC metrics and indicating robust generalization capabilities. This method effectively mitigates the noise problem inherent in distant supervision data, thereby enhancing the overall performance of relation extraction tasks. The effectiveness of the hierarchical contrastive learning model is further validated using the denoised dataset, confirming its ability to improve relation extraction accuracy and reliability.

## Data Availability

Due to institutional data privacy policies, the dataset generated in this study is not publicly available, but can be obtained from the corresponding author upon reasonable request. If you need data, you can contact the corresponding author: aygul1212@stu.xju.edu.cn.
